# Prognostic and predictive role of CXCR4, IGF-1R and Ezrin expression in localized synovial sarcoma: is chemotaxis important to tumor response?

**DOI:** 10.1186/s13023-014-0222-5

**Published:** 2015-01-23

**Authors:** Emanuela Palmerini, Maria Serena Benassi, Irene Quattrini, Laura Pazzaglia, Davide Donati, Stefania Benini, Gabriella Gamberi, Marco Gambarotti, Piero Picci, Stefano Ferrari

**Affiliations:** PROMETEO Laboratory/Section of Chemotherapy, Research, Innovation & Technology (RIT) Department, Istituto Ortopedico Rizzoli, Via Pupilli, 1, 40136, Bologna, Italy; Laboratory of Experimental Research, Bologna, Italy; Orthopaedic Surgery, Bologna, Italy; Surgical Pathology, Bologna, Italy; Section of Chemotherapy Musculoskeletal Oncology Department, Istituto Ortopedico Rizzoli, Bologna, Italy; Department of Biomedical and Neuromotor Sciences, University of Bologna, Bologna, Italy

**Keywords:** CXCR4, IGFR1, Ezrin, Synovial sarcoma, Prognostic factors, Predictive factors

## Abstract

**Background:**

Synovial sarcoma (SS) is a rare tumor, with dismal survival when metastatic. The role of adjuvant chemotherapy is debated. New prognostic and predictive factors are needed.

**Methods:**

We reviewed patients with localized SS; SS18-SSX fusion transcript presence was confirmed by FISH and RT-PCR. Expression of CXCR4, IGF-1R and Ezrin were evaluated by immunohistochemistry.

**Results:**

Tumor samples from 88 SS patients (45 female; 43 male) with median age 37 years (range 11–63) were selected. The size of the lesion was > 5 cm in 68% of patients and 34% of cases presented biphasic histotype. All patients underwent surgery, 56% adjuvant radiotherapy (RT), 65% adjuvant chemotherapy. A positive stain for IGF-1R was detected in 55 patients, with nucleus expression in 21 patients. CXCR4 was expressed in 74 patients, nuclear pattern in 31 patients. 80 SS were positive to Ezrin, 48 had cytoplasmatic location, 32 membrane location. With a median follow-up of 6 years (1–30 years), the 5-year overall survival (OS) was 70% (95% CI 60–81). 5-year OS was 63% (95% CI 41-85%) for patients with positive IGF-1R/nuclear expression, and 73% (95% CI 61-85%; *P* = 0.05) in negative patients. 5-year OS was 47% (95% CI 27-66%) in patients with positive CXCR4/nuclear staining, and 86% (95% CI 76-96%, *P* = 0.0003) in negative cases. No survival difference was found according to Ezrin expression. By multivariate analysis, nuclear expression of CXCR4 and IGF-1R was confirmed independent adverse prognostic factor for SS patient survival linked to the use of chemotherapy.

**Conclusions:**

Our findings have important potential implications demonstrating that together with clinical prognostic factors such as radiotherapy and age, CXCR4 and IGF-1R negatively influences survival in patients with localized SS. We believe that further studies addressed to the effects of CXCR4 and IGF-1R inhibitors on cell viability and function are needed to plan new and more appropriate SS treatments.

## Background

Synovial sarcoma (SS) comprises approximately 8% of all soft tissue sarcomas (STSs), with the lower limbs being the most common site of primary disease [1]. Although relatively rare, SS is the third most common extremity STS. It affects mostly young adults, with a median age of 35 years [2]. Three histologic subtypes of SS are described: monophasic, entirely composed of spindle cells; biphasic, composed of both spindle cells and epithelial cells; and poorly differentiated subtypes [3]. Synovial sarcoma contains a characteristic translocation (X;18)(p11;q11), representing the fusion of *SYT* on chromosome 18 with either *SSX1, SSX2,* or rarely *SSX4* on chromosome X [4]. The resulting fusion genes appear to be mutually exclusive and concordant in primary and metastatic tumors [5]. In a previous series of 250 patients with SS dating back to 1976, we demonstrated that stage, size, age, and histologic subtype were independent factors for event free survival [6]. Also, this study provided further evidence that adjuvant radiotherapy is a significant independent prognostic factor, and should always be performed in large lesions [7]. Other factors, such as surgical margins, p53 overexpression, Ki-67 proliferative index, and *SYT-SSX* fusion type, have been identified [8-13]. The role of adjuvant chemotherapy in SS is debated. In the metastatic setting, a high response rate to ifosfamide-based therapy has been reported (40%-70%) [14,15]. Therefore, adjuvant chemotherapy is frequently used for localized disease [1,16]. A previous genetic study [17] identified a hypoxia-induced metastatic profile in pleomorphic high-grade STSs providing information for selection of high-risk tumors. Multiple regulators of signalling pathways including EGF and FGF receptors, members of the Hedgehog (Hh) family, genes involved in retinoic and Notch pathways, and in chromatin remodelling were found up regulated in SS [18].

Chemokine receptor 4 (CXCR4) is a seven-transmembrane G protein-coupled chemokine receptor and it is the chemokine receptor most commonly expressed in tumor cells, with increased expression in presence of metastatic disease in many tumors including bone and soft tissue sarcomas [19,20]. CXCR4 has also been demonstrated to be involved in cell migration and invasion, as well as angiogenesis.

Insulin growth factor-1 receptor (IGF-1R) is involved in IGF-II signalling and down-regulation or inhibition of this receptor leads to increased numbers of apoptotic cells in SS18–SSX-transformed cells and SS cell lines [21]. In addition, a study that investigated IGF-1R expression in 35 SS found that there was an association between IGF-1R expression and an increased incidence in lung metastasis [22]*.*

Ezrin is a membrane-cytoskeleton linker protein involved in growth regulating and metastatic behaviour of cancer cells. Our previous experience in osteosarcoma detected Ezrin immunoreactivity in the majority of patients with non-metastatic osteosarcoma of the extremity and revealed that the cytoplasmatic pattern was associated with good prognosis [23].

In order to identify a subgroup of patients with poorer prognosis who most likely benefit from adjuvant chemotherapy, we selected localized chemo-naïve SS patients from our previously studied series [6] and assessed the prognostic role of CXCR4, IGF-1R and Ezrin by correlating their expression with clinical and histological parameters.

## Methods

### Design and patients

This study is a systematic mono institutional retrospective analysis. From the previous series of 250 patients [6] referred to our Institute between 1976–2008, we selected patients admitted for first diagnosis; therefore, metastatic and recurrent patients at presentation were excluded. All patients with incomplete clinical and follow-up data were also excluded. General informed consent to the use of material was obtained from all adult patients or from parents/guardians for minors from 2004.

The research protocol was approved by the ethics committee of the Rizzoli Orthopedic Institute.

### Data collection

Demographic data (age at onset, gender, follow-up duration), clinical-histological presentation (biphasic, monophasic/poorly differentiated, tumor size, site), treatment (R0/R1 resection, adjuvant chemotherapy, adjuvant radiotherapy), and outcome (overall survival (OS), were collected. Follow-up was obtained from hospital charts, or if necessary, by a phone call.

### Histology and molecular studies

The diagnosis was confirmed by pathologists with expertise in soft tissue and bone tumors, after revision of histological slides according to histopathological and immunohistochemical criteria [3]. The presence of SS18 (SYT) gene rearrangement and fusion transcripts was assessed by Fluorescence in situ hybridization (FISH) and Reverse Transcriptase-Polymerase Chain Reaction (*RT*-PCR) analysis for diagnosis confirmation.

### FISH analysis

FISH was performed using the SS18(SYT) (18q11.2) VYsis LSI Dual Color Break-apart DNA probes (Abbott Molecular, Des Plaines, IL, USA) according to the manufacturer’s protocol. A minimum of 100 tumor cell nuclei with intact morphology as determined by DAPI counterstaining were counted in the previously marked neoplastic area. A positive result was defined as the presence of a visible translocation (separation of red and green signals > 3 signal diameters) in more than 10% of the cells [24].

### RT-PCR analysis

RNA was extracted from fresh tissue using a modified method including Trizol Reagent (Invitrogen, Carlsbad, CA) and the column of RNeasy Mini Kit (Qiagen GmbH, Hilden Germany). 2 μg of RNA was reverse transcribed using High Capacity cDNA Archive Kit (Applied Biosystems, Foster City, CA). The primers used for detection of t(X;18) SS18(SYT)-SSX1 and t(X;18) SS18(SYT)-SSX2 by PCR amplification was: forward SS18 5′GGA CAA GGT CAG CAG TAT GGA3′; reverse primers for SSX1 5′TTG GGT CCA GAT CTC TTA TT3′; and reverse for SSX2 5′TTGGGTCCAGATCTCTCGTG3′ [25].

### Immunohistochemistry staining

Paraffin embedded tissue from non-treated patients was used for immunohistochemistry *(*IHC) analysis. The sections (4 μm thickness) were de-paraffinized by 30 minutes in dry oven at 55-60°C and 1 hour in xylene; then the slides were rehydrated in graded alcohol 100%, 95%, 75%. After rinsing with water for 2 minutes, sections were pre-treated with 3% hydrogen peroxide for 5 minutes and Universal Block (KPL, Inc.) for 15 minutes. The sections were incubated for 30 minutes at room temperature with monoclonal antibodies CXCR4 (Ab-2074, Abcam, Cambridge, UK; 1:1000 dilution), IGF-1R (C-20 Santa Cruz Biotechnology, Inc. CA, USA; 1:100 dilution), Ezrin (ThermoScientific, Fremont, CA, USA; 1:200 dilution).

Antibody detection was performed using UltraView Universal DAB Detection Kit and UltraView Universal Alkaline Phosphatase Red Detection Kit (Ventana Medical Systems, Tucson AZ, USA). Pre-treatment for antigen retrieval was performed at 95°C with Tris-EDTA ph 8 for 20 minutes. Sections were counterstained with haematoxylin, dehydrated and mounted.

The negative control was prepared omitting the primary antibody. Positive controls included in each run were HELA for CXCR4 and human kidney for IGF-1R and Ezrin.

The immunoreactivity was interpreted by the percentage of positive cells (0 = negative, 1 = <25% of cells, 2 = 26-75% of cells, 3 = positivity of >75% of cells). All samples scored 1 to 3 were considered positive. Cytoplasmatic, nuclear and membrane immunoreactivity was considered for the staining distribution pattern.

### Statistical analysis

The following parameters were examined for statistical prognostic correlations: patient age, tumor size, surgical margins, histology, use of chemotherapy, use of radiotherapy, total expression of CXCR4, nuclear and/or membrane/cytoplasm expression; total expression of IGFR1, nuclear and/or cytoplasmatic expression. Total expression of Ezrin, cytoplasmatic only and cytoplasmatic/membrane expression. The following categories were compared: patients age (adolescent and young adults (AYA) with < 30 years versus adults with ≥30 years); tumor size (≤5 cm versus >5 cm); surgical margins (adequate including wide or radical versus inadequate including intralesional, marginal or contaminated margins, according to Enneking’s classification) [26]; histology (biphasic versus other histotypes); adjuvant treatments (chemotherapy or radiotherapy performed within 3 months after tumor excision). Chi-square (χ^2^) test with Fisher’s exact p value was used to correlate protein expression with clinical parameters. Overall survival (OS) time was calculated from the time of admission at our Institute to death or last follow-up visit. All time-to-event end points were modelled using the method of Kaplan and Meier and analysed by the log-rank test. The results of the Cox model analysis were reported as relative risks (RRs) and 95% confidence intervals (CIs).

## Results

A total of 88 consecutive patients were selected. Forty-five were female and 43 male; median age was 37 years (range 11–63); 14 were adolescents and young adults (AYA) ( <30 years) and 74 were adults (≥30 years). Size of the lesion was > 5 cm in 60 patients (68%), ≤ 5 cm in 24 (27%) and unknown in 4 patients. The tumor site was trunk in 13 cases (15%), lower extremity in 64 cases (73%), upper extremity in 11 cases (12%). Concerning histotypes, 30 patients (34%) had biphasic SS, 51 (58%) monophasic SS and 7 (8%) poorly differentiated SS. All patients underwent surgery with adequate surgical margins in 68 cases, inadequate in 18 and in 2 cases surgical margins were unknown. Amputation was performed in 24 patients (27%). Forty-seven patients (53%) underwent adjuvant radiotherapy (RT) and 57 (65%) chemotherapy, 27 of them (47%) preoperatively (epirubicin/adrymicin and ifosfamide combination in 49 cases, non-ifosfamide containing regimen in 8 cases and in 1 case the treatment was unknown).

### FISH and RT-PCR Analysis

By FISH analysis, all 88 SS presented SS18 (SYT) gene rearrangement resulting in (X;18)(p11.2;q11.2) translocation, thus confirming the histological diagnosis. The presence of SSX fusion transcripts was assessed on 46 frozen tissues by *RT*-PCR: 28 cases presented SSX1 variant, 18 had SSX2 variant.

### Immunohistochemistry

**Figure 1:**
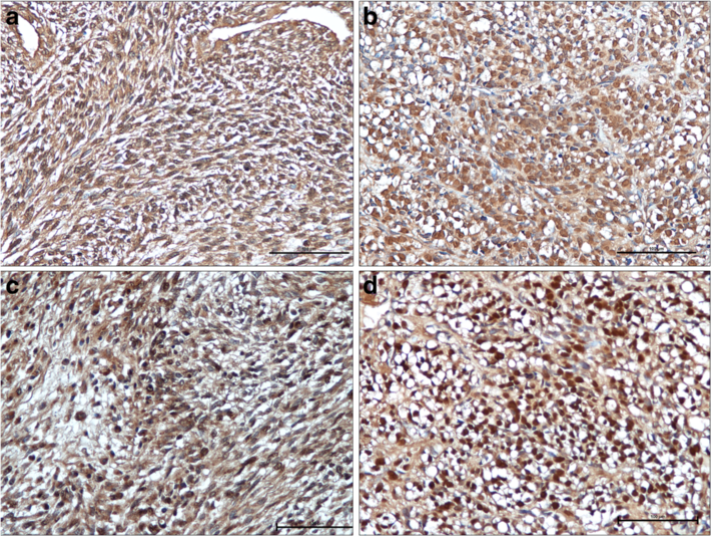
**Expression of CXCR4 and IGF-1R in synovial sarcoma.** Immunohistochemical expression of CXCR4 with cytoplasmatic **(a)** and nuclear **(b)** distribution. Immunohistochemical expression of IGF-1R with cytoplasmatic **(c)**, and nuclear distribution **(d)** (IHC 20X).

CXCR4 was positive in 74 SS (84%), 43 had a membrane/cytoplasmatic staining, 31 nuclear (7 nuclear only and 24 nuclear and cytoplasmatic) (Figure 1a,b).

A positive expression for IGF-1R was detected in 55 patients (62.5%), 34 had a membrane/cytoplasmatic staining, 21 presented nuclear positivity (5 nuclear only and 16 both nuclear and cytoplasmatic) (Figure 1c,d). No significant difference was observed for CXCR4 and IGF-1R proteins expression between biphasic and monophasic SS in terms of positivity [CXCR4: biphasic: 25/30 (83%); monophasic: 44/51 (86%); IGF-1R: biphasic: 17/30 (57%); monophasic: 33/51 (64%)], and intracellular distribution [nuclear CXCR4: biphasic: 8/30 (27%); monophasic: 21/51 (41%); IGF-1R: biphasic: 6/30 (20%); monophasic: 14/51(27%)].

Nuclear expression of both CXCR4 and IGF-1R was detected in 8 cases.

80 SS were positive to Ezrin (91%), 48 presented only cytoplasmatic location while 32 had cytoplasmatic and/or membrane staining. Interestingly, the percentage of positive cases in biphasic was higher than in monophasic subgroup (100% vs. 86%, exact Fisher’s test *P* = 0.04). Concerning intracellular staining distribution, while biphasic had exclusively Ezrin cytoplasmatic location in about half of cases (16/30; 53%) and combined cytoplasmatic/membrane expression in 14 /30 (47%), the distribution pattern of monophasic SS was predominantly cytoplasmatic. In detail, 32/44 cases (73%) had only cytoplasmatic staining, 12/44 (27%) had both cytoplasmatic and membrane positivity and 6 cases had localized membrane reactivity.

### Outcome and statistical correlations

With a median follow-up of 6 years (1–30 years), the 5-year overall survival (OS) was 70% (95% Cl 60-81%). The 5-year OS was significantly better for young patients (100% for AYA and 65% for adult patients, *P* = 0.003).

For CXCR4 and IGF-1R expression and outcome correlation, the positivity in the nucleus was chosen and defined as CXCR4/nuclear staining and IGF-1R/nuclear expression.

**Figure 2:**
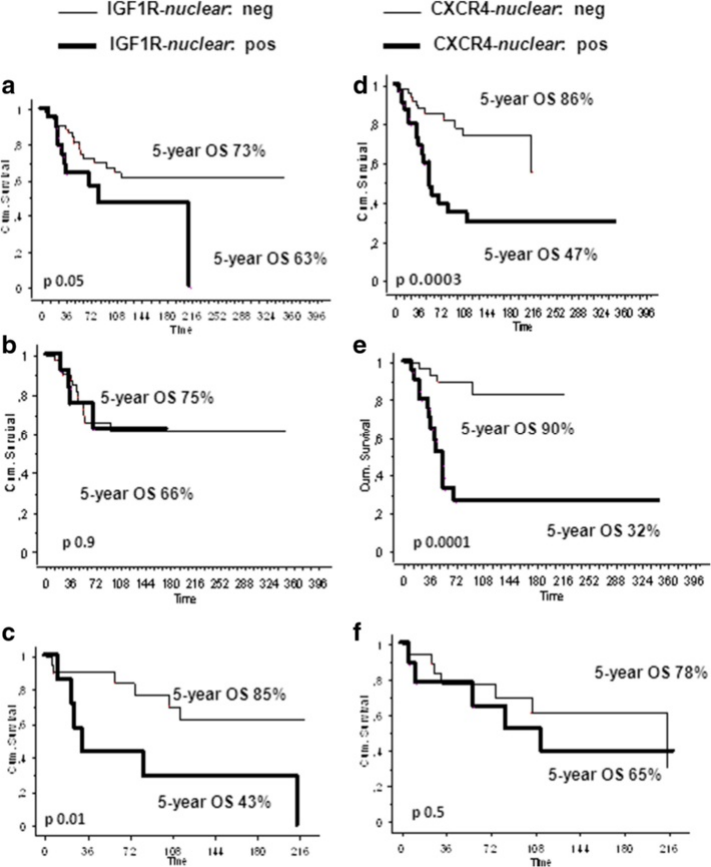
**Overall survival curves according to nuclear IGF-1R (a, b, c) and CXCR4 (d, e, f) expression (negative versus positive) for (a, d) the whole population (n = 88), (b, e) for the patients who did receive an adjuvant treatment (n = 60), (c, f) for the patients who did not receive an adjuvant treatment (n = 30).**

The 5-year OS was significantly better for patients with negative CXCR4/nuclear staining (86% for negative and 47% for positive patients, *P* = 0.0003) (Figure 2d), and for patients with negative IGF-1R/nuclear expression (73% for negative and 63% for positive patients, *P* = 0.05) (Figure 2a). According, combined CXCR4/IGF-1R/nuclear positive staining (*double positive*) was associated with poorer survival (*double positive*: 5-year and 8-year OS 57% (% CI 20–94) and 20% (% CI 0–52); non- *double positive:* 5-year and 8-year OS 71 (% CI 60–83) and 67% (% CI 55–73); *P =* 0.02).

No significant correlation between Ezrin expression and clinical variables was found (Table 1).

**Table 1:** **Univariate analysis for 5-year OS**

**Variable**		**Patient N° (88)**	**5-year OS (%)**	**95% CI**	**p**
**Age**	Adult	74	65	53-77	0.003
	AYA	14	100		
**Size**	<5 cm	24	82	66-98	0.4
uk in 4 pts	≥5 cm	60	67	53-80	
**Margins°**	Adequate	68	69	57-81	0.8
	Inadequate	18	76	51-100	
**Histology**	Biphasic	30	67	53-80	0.5
	Other	58	76	59-93	
**Chemotherapy**	No	28	73	67-85	0.5
	Yes	60	68	54-81	
Radiotherapy*	No	37	67	51-83	0.07
	Yes	47	77	63-92	
**IGF-1R**	Negative	33	72	53-90	0.3
	Positive	55	69	56-82	
**IGF-1R/nuclear**	Negative	67	73	61-85	0.05
	Positive	21	63	41-85	
**CXCR4**	Negative	14	92	76-100	0.1
	Positive	74	66	54-78	
**CXCR4/nuclear**	Negative	57	86	76-100	0.0003
	Positive	31	47	54-78	
**EZRIN**	Negative	8	100		0.2
	Positive	80	67	55-78	
**EZRIN positivity pattern**	Cytoplasmatic	49	65	50-79	0.9
	Cyto/membrane	31	70	52-89	

AYA: adolescent and young adults; OS: overall survival; IGF-1R: insulin growth factor-1 receptor; CXCR4: chemokine (C-X-C motif) receptor 4; uk:

**°**unknown in 2 pts.

*unknown in 4 pts.

An increased overall survival, close to statistical significance (p = 0.07), was documented in patients undergoing radiation therapy, while chemotherapy, surgical margins, histologic subtype (biphasic vs. other) as well as Ezrin expression did not have any impact on the outcome (Table 1).

Furthermore, in 46 patients with frozen tissues available we found no difference between SSX variants. The 5-year OS was 74% (% CI 56–92) for SSX1, and 68% (% CI 42–94; p = 0.8) for SSX2.

Finally, we performed a survival analysis in 2 subgroups of patients: chemotherapy *treated* and *untreated* patients. In the first group the 5-year OS was 75% for patients with IGF1R/nuclear negative staining versus 66% for positive patients (p = 0.9) and 90% for patients with CXCR4/nuclear negative staining versus 32% for positive patients (p = 0.0001) (Figure 2b,e). In the group of *untreated* patients, the 5-year OS was and 85% for patients with IGF1R/nuclear negative staining versus 43% for positive patients (*P* = 0.01) and 78% for patients with CXCR4/nuclear negative staining versus 65% for positive patients (*P* = 0.5) (Figure 2c,f). After multivariate analysis nuclear expression of CXCR4, IGF-1R and use of RT were confirmed statistically significant independent factors for OS, while and age were not (Table 2).

**Table 2:** **Multivariate analysis for 5-year OS**

**VARIABLE**		**HR for 5-year OS**
**IGF-1R-nuclear**	Positive	1
	Negative	0.4 [0.2-0.9]
		p = 0.04
**CXCR4-nuclear**	Positive	1
	Negative	0.3 [0.1-0.7]
		p = 0.003
**SIZE**	≤5 cm	1
	>5 cm	2.6 [1–7]
		p = 0.06
**RADIOTHERAPY**	Yes	1
	No	3.8 [1.6-9.2]
		p = 0.002
**AGE**	AYA	1
	Adult	6.3 [0.8-50.2]
		p = 0.08

HR: hazard ratio; AYA: adolescent and young adults; OS: overall survival; IGF-1R: insulin growth factor-1 receptor; CXCR4: chemokine (C-X-C motif).

## Discussion

As for other high-grade malignant soft tissue tumors, the standard treatment of SS is the wide surgical removal of the lesion and radiotherapy [7]. Survival rate ranges from 62% to 83% in a variety of studies and better results are reported for smaller tumors (<5 cm), and for those in which negative surgical margins are achieved [6,16].

The role of adjuvant chemotherapy in SS remains controversial and definitive conclusions have been difficult to make in absence of histology-specific chemotherapy protocols. Nonetheless adjuvant chemotherapy has been employed in case of localized disease, especially in the pediatric population [12]. With the currently available treatments, in particular in adults and for advanced/recurrent disease [15,27], the prognosis of SS remains unsatisfactory and there is an urgent need to identify prognostic and predictive factors.

Our SS population is representative of the largest previously published SS series [6,12] for clinical and treatment characteristics and, most importantly, for expression of prognostic factors for overall survival. This study includes a consecutive series of localized SS patients, treated in a referral center. In all cases the diagnosis of SS was confirmed by the presence of (X;18)(p11.2;q11.2) translocation [13] and the possible prognostic role of IGF-1R, CXCR4 and Ezrin was assessed by correlating protein immunoreactivity with clinical and histological features.

Nuclear expression of CXCR4 and IGF-1R resulted a strong independent adverse prognostic factor for overall survival. Interestingly, the meaning of the nuclear expression of the two markers was linked to the use of chemotherapy. In fact, IGF-1R/nuclear expression was significantly related to a poor probability of survival, but only in patients who did not undergo adjuvant chemotherapy. On the contrary, CXCR4/nuclear negative expression was predictive of poor prognosis, but only in patients who received adjuvant chemotherapy.

IGF-1R is a transmembrane receptor highly expressed in many human cancers, including sarcomas [28]. The implication of IGF/IGF-1R axis in SS development and management was discussed in a study demonstrating that SS18–SSX1 or SS18–SSX2 fusion genes up-regulate insulin-like growth factor-2 (IGF-2) through epigenetic mechanisms [29]. Finally, by microarray analysis, high expression levels of the ligand IGF-2 were found in SS samples [30], that also expressed IGF-1R [31]. In this study we demonstrated that a high percentage of SS presented positivity for IGF-1R, with nuclear and/or cytoplasmatic immunostaining in both monophasic and biphasic subtypes. As previously described, IGF-IR auto-regulates IGF-IR gene by translocating to nucleus [32].

In our SS, as in breast and lung tumor [32,33], the survival was significantly better for patients with negative IGF-1R/nuclear expression when compared to nuclear-positive patients. Although this result emphasizes the importance of immunostaining location, the poor-prognostic significance of IGF-1R/nuclear expression, also confirmed by multivariate analysis, was limited to a subgroup of patients who did not receive systemic therapy. We believe that this may reinforce the role of IGF-1R as strong prognostic factor in SS, selecting high-risk patients candidates for adjuvant therapy.

Recently, Asmane et al. [34] suggested that the nuclear location of IGF-1R might activate signaling pathways demonstrating that patients with advanced sarcoma had better progression-free and overall survival when treated with IGF-1R antibody therapy.

In the last years there is an increasing interest on the interaction of the cancer cells with their microenvironment, mediated by the chemokine ligand CXCL12, and its chemokine receptor 4 (CXCR4) [35]. This axis plays critical roles in tumor progression, including promotion of tumor cell proliferation and survival, metastasis and angiogenesis [36]. CXCR4 is important for the prognosis of several tumor types, including melanoma [36], colon [37], pancreatic [38], gastric [39] cancer and STS [20]. Our results showed that CXCR4 nuclear expression was an independent adverse prognostic factor for localized SS predominantly in the group of patients who received chemotherapy. Accordingly, D’ Alterio et al. [40] demonstrated that a high expression of CXCR4 was associated with poor response in metastatic renal cancer patients treated with sunitinib.

Thus, the inferior survival of SS patients with CXCR4 nuclear positivity, as compared with negative patients, might suggest that CXCR4 is involved in mechanisms of resistance to chemotherapy. The anti-tumor activity of a CXCR4 antagonist has been shown in pre-clinical and animal tumor models [41], and several clinical studies on CXCR4 antagonists as chemosensitizer for treatment of patients with hematological and solid tumors are underway [35,42].

The role of Ezrin, a cytoskeleton linker protein that is actively involved in regulating the growth and metastatic capacity of cancer cells, has been reported in adult soft tissue sarcoma with a direct correlation between IHC staining intensity, histological grade and infiltrative growth pattern [43]. In our series the majority of SS were positive to Ezrin revealing that expression and distribution pattern of staining (cytoplasmatic/membraneous) was not as relevant for prognosis as in osteosarcoma [23]. However, all 8 patients with negative Ezrin immunostaining were alive at 5 years.

Altogether, these findings, should be analyzed in the context of older reported prognostic factors such as Ki67 and p53 [44,45], and newer ones such as CINSARC [46]. In a future scenario an high expression of Ki67, a mutated p53, a C2 CINSARC signature (increased genomic complexity), together with nuclear positivity for CXCR4 and IGF-1R expression, could represent novel tools to stratify SS patients for treatment.

## Conclusions

Our findings have important potential implications demonstrating that together with clinical prognostic factors such as radiotherapy and age, CXCR4 and IGF-1R nuclear expression is a strong independent adverse prognostic factor for SS patient survival, linked to the use of chemotherapy. Based on these data, CXCR4/nuclear expression was predictive of poor prognosis in patients who received adjuvant chemotherapy, emphasizing its possible involvement in drug-resistance mechanisms. In contrast, IGF-1R/nuclear expression, significantly related to poor survival in patients who did not receive adjuvant chemotherapy, may differentiate a subgroup of SS patients candidate to adjuvant chemotherapy.

We believe that CXCR4 antagonists, combined with chemotherapy, could act as chemosensitizer in SS, as suggested in other hematological and solid tumors settings [35,42]. Further studies addressing the effects of CXCR4 and IGF-1R inhibitors on cell viability and function are needed to plan new and more appropriate SS treatments.
